# A Coroner and His Jury

**Published:** 1901-05-04

**Authors:** 


### A Coroner and his Jury.

A curious case is recorded as having occurred in the court of Mr. E. Wynne Baxter, Coroner for the Eastern division of the County of London, during an inquest upon one Andrew Walsh, late a "paster" in the employment of the Electric Storage Company, concerning whom the coroner said that it had been "suggested" (by whom did not appear) that he had died from lead poisoning. "A post-mortem examination had been made without his (the coroner's) knowledge, and a portion of the body had been carried away. A certificate was given, stating that death was due to intestinal obstruction. That might be correct, but death might *also* arise from lead poisoning. In all cases where lead poison was suspected or suggested it was the duty of the medical man to report the same to the coroner, so that the case might be properly investigated."

It appeared from the evidence that the deceased was taken ill on his return from work on the evening of Tuesday, April 16th, with severe abdominal pain and vomiting. He was seen that night by Dr. Moffat, assistant to Dr. Wilson, and on the following morning by Dr. Wilson himself, and the nature of the case seems to have been obscure. He died on Thursday morning, the 18th; and Dr. Wilson, for his own satisfaction, obtained permission to make a post-mortem examination, which, according to the report in the *St. James's Gazette*, disclosed intestinal obstruction from a malignant growth. The doctor gave a certificate in accordance with this finding, and the poor man would have been buried in the ordinary course, had it not been for the fact that his work required him to handle lead, and that some wiseacre accordingly suggested that he had fallen a victim to lead poisoning. On this very feeble ground, and in total disregard of a medical certificate resting upon observation during life as well as upon an autopsy, the coroner thought fit to hold an inquest, in the course of which he had what we can only describe as the impudence to censure Dr. Wilson for having made a post-mortem examination on his own responsibility, and without first "consulting" him (the coroner) on the subject. Dr. Wilson was, of course, perfectly within his rights in making the post-mortem, although by doing so he lost the fee which might otherwise have come to him; and his discovery of a manifest and sufficient cause of death not only removed all possible grounds of "suspicion" from the case, even if, in his mind, any such had existed, but also rendered it his statutory duty to give a certificate in accordance with the facts. If the coroner is correctly reported as having said that in all cases where lead poisoning was "suspected or suggested" it was "the duty of the medical man to report the case to the coroner," he was simply talking outrageous nonsense. The first question to arise would be: Suspected by whom? Suggested by whom? Dr. Wilson probably knew that the symptoms bore no resemblance to any which lead poisoning would be calculated to produce, and that the suggestion or suspicion was simply foolish, so that there could be no moral claim upon him to take any action on account of it. Legal claim there could be none, because communication with a coroner forms no part of the duty of a medical man, even in cases in which he may entertain suspicion himself. His duty terminates with his refusal to certify the cause of death, and the duty of communicating with the coroner appertains to the registrar of deaths and not to the doctor. Sometimes, no doubt, the doctor does communicate with the coroner; but in doing so he is acting as a simple citizen in the interests of the security of life, and not as a member of the medical profession, or in discharge of any claim which can be made upon him in his capacity as a medical practitioner. The jury were not slow to improve upon the position assumed by their presiding official, and instead of the verdict in accordance with the evidence which the law is supposed to require, they returned one in direct opposition to it. The words of this extraordinary deliverance, as published by our contemporary, are:—"That apart from the medical evidence given, we are of opinion that the deceased died from lead poisoning, and that such death was due to misadventure." Were the words "apart from" intended to mean "notwithstanding, or in spite of?" or were they intended to mean that if the jury had not had the guidance of medical evidence to the contrary, they might have arrived at the mistaken opinion indicated? We only know of a single parallel verdict, and it was given by an Irish jury in a case in which Daniel O'Connell defended a man accused of murder. The man said to have been murdered was produced in court, and swore that he had never seen the prisoner. The "Liberator" used to finish the story by saying "I confidently expected an acquittal; but party spirit ran very high in the barony, and the jury found the man guilty of horse stealing." In all probability, something analogous to party feeling may exist in Poplar with regard to the effects of lead upon those who work in it. However this may be, it is to be hoped that the Government, whenever they find time to improve the present faulty system of certifying and registering the causes of death, may be able to protect medical practitioners and the public against the ignorance and presumption of coroners.

